# Hypoxia-Induced ZWINT Mediates Pancreatic Cancer Proliferation by Interacting With p53/p21

**DOI:** 10.3389/fcell.2021.682131

**Published:** 2021-11-24

**Authors:** Peng Chen, Zhiwei He, Jie Wang, Jian Xu, Xueyi Jiang, Yankun Chen, Xinyuan Liu, Jianxin Jiang

**Affiliations:** ^1^ Department of Hepatobiliary Surgery, Renmin Hospital of Wuhan University, Wuhan, China; ^2^ Department of Hepatic-Biliary-Pancreatic Surgery, The Affiliated Hospital of Guizhou Medical University, Guiyang, China

**Keywords:** ZWINT, pancreatic cancer, HIF1α, p53/p21, proliferation

## Abstract

p53/p21 signaling plays a vital role in pancreatic cancer (PC) progression. ZWINT was shown to function as an oncoprotein in the progression of multiple cancers. However, the involvement of ZWINT and p53 activation in the progression of PC remains poorly understood. Bioinformatics and tissue array chip analyses were performed to evaluate ZWINT expression in pancreatic cancer. ZWINT mRNA and protein expression were evaluated in normoxia and hypoxia. CHIP was used to evaluate HIF1α interaction with the ZWINT promoter. CCK8, colony formation, EDU, and cell cycle analysis were used to examine PC cell proliferation. Immunoprecipitation and immunofluorescence were used to examine the interaction of ZWINT, MDM2, and p53. p53 activity was evaluated by q-PCR and luciferase assay. Protein degradation and ubiquitination assays were used to analyze the role of ZWINT in p53 ubiquitination. ZWINT was overexpressed in pancreatic cancer and induced in hypoxia. ZWINT promoted pancreatic cancer growth and cell cycle progression. Bioinformatic analysis revealed that ZWINT may regulate the p53 signal pathway. ZWINT interacts with p53 and promotes its ubiquitination and degradation. ZWINT promoted proliferation via p53/p21. Immunohistochemistry of clinical specimens revealed that that ZWINT expression was significantly negatively correlated with p53/p21. Our data showed that hypoxia regulates the expression of ZWINT, which activated p53/p21 signaling pathway to promote PC growth.

## Introduction

Pancreatic cancer (PC), one of the most serious gastrointestinal malignancies, is the fourth most frequent cause of cancer-associated death (Siegel et al., 2020). Because of the lack of precise diagnostic approaches, PC is usually diagnosed at advanced stage (The Lancet Gastroenterology and Hepatology, 2021). Patients with advanced PC can only receive chemotherapy instead of surgical resection (Perri and Katz, 2021). In the occurrence and development of pancreatic cancer, there are no more accurate early diagnosis and treatment methods. (Lee et al., 2020). Hence, studies of diagnostic markers and targeted therapies that inhibit the progression of PC have been key strategies to improve the treatment of PC.

ZW10-interacting kinetochore protein 1 (ZWINT-1) is a centromere complex component necessary for the mitotic spindle checkpoint and participates in centromere function and cell development (Peng et al., 2019). ZWINT interacts with Zeste White 10 (ZW10), another centromere protein, to possibly regulate the relationship between ZW10 and centromeres (Woo et al., 2015). ZWINT-1 is a kinetochore component with a significant role in spindle assembly and kinetochore-microtubule attachment during meiosis and mitosis (Kasuboski et al., 2011). In addition, ZWINT-1 directly interacts with components of the KMN complex, specifically Ndc80 and Mis12, and functions as a bridge between the RZZ and KMN complexes necessary for kinetochore formation and spindle checkpoint activity (Zhang et al., 2015). As a mitotic checkpoint component, ZWINT-1 is required for the stable relationship between CENP-F and dynamitin and the kinetochore to guarantee precise chromosome segregation (Zhang et al., 2015).

Recent studies have indicated that ZWINT-1 may function as a biomarker for cancer due to its high expression in some human malignancies such as glioblastoma (Yang et al., 2020), breast cancer (Zhou G. et al., 2020), ovarian cancer (Zhao and Yu, 2020), bladder cancer, lung cancer (Yi et al., 2020), and hepatocellular carcinoma (Xie et al., 2020). In addition, knockdown of ZWINT-1 restrained the proliferation, migration, invasion, and colony formation abilities of pancreatic cancer cells and increased cell apoptosis (Kim et al., 2020). Another study showed that ZWINT interacts with Rab3C as a part of the MIS12 complex (Obuse et al., 2004). Endo et al. showed that cell proliferation in 293T cells and breast cancer MCF7 cells (Endo et al., 2012) were negatively regulated by ZWINT depletion by Terf/Trim (Endo et al., 2012). Together these findings suggest that ZWINT, which plays a role in the spindle filament and cell division, may function as an oncoprotein. However, the role of ZWINT in pancreatic cancer has remained unknown.

It was found that the aberrantly of ZWINT protein expression promoted PC pathogenesis as was a distasteful element. In addition, ZWINT promoted p53 ubiquitin degradation of p53 via interacting with MDM2, inactivating p53/p21 signaling, promoting the transition of cell cycle and cell development in PC. Our findings indicate that ZWINT functions as an oncoprotein and modulates p53/p21 to promote pancreatic cancer pathogenesis.

## Materials and Methods

### Specimen Source and Clinical Materials

This study included 92 pancreatic cancer samples, including tumors and adjacent non-tumor tissues. The pancreatic cancer tissues were obtained from surgical specimens from inpatients in our hospital from 2016 to 2020.

### 5-Ethynyl-2′-Deoxyuridine Assay

Cells in a 24-well plate were incubated with 200 μl of EDU medium (5 μM) for 2 h and then fixed with 50 μl phosphate buffer saline (PBS) containing 4% paraformaldehyde for 30 min at room temperature. Cells were then incubated with 50 μl of glycine (2 mg/ml) for 5 min, followed by a 10-min treatment of 200 μl of osmotic agent (PBS containing 0.5% Triton X-100), a 30-min treatment of 200 μl of IX Apollo staining solution at room temperature in the dark, and two to three rinses with 200 μl of osmotic agent (PBS containing 0.5% Triton X-100; 10 min each time). Cells were counterstained with 4′, 6 diamidino-2-phenylindole (DAPI) for 5 min for nuclear staining and then examined using a fluorescence microscope.

### Luciferase Reporter Assays

It is predicted that the human DNA sequences of the p21 promoter are bound with transcription element p53 cloned into a pGL3 vector. Cells were transfected with luciferase plasmids along with ZWINT or control lentivirus for x h. Cell lysates were obtained and luciferase activity was detected using the dual-luciferase reporter system according to the manufacturer’s instructions (Promega, Madison, WI, United States).

### RT-PCR

RNA was extracted from cells using Trizol based on the manufacturer’s instructions, and a UV spectrophotometer was used to determine the purity and concentration of the RNA. RNA was reverse transcribed into cDNA using the RT-PCR kit TAKARA047A (Takara Bio, Inc., Shiga, Japan) of the Super Script III First-Strand Synthesis System. The BioRad Real-Time PCR system was used for real-time PCR amplification. The primers for ZWINT, p53, p21, CDK4, CDK6, Cyclin D1, Cyclin E1, and GAPDH mRNAs are shown in Table 1.

**TABLE 1:** The RNA primers used in the PCR.

Gene name	Sequences
ZWINT (NM_001005413)	F:AGGACACTGCTAAGGGTCTCG
	R:GCCTCTACGTGCTCCCTGTA
P53 (NM_001126118)	F:ACAGCTTTGAGGTGCGTGTTT
	R:CCCTTTCTTGCGGAGATTCTCT
P21 (NM_078467)	F:TGTCCGTCAGAACCCATGC
	R:AAAGTCGAAGTTCCATCGCTC
CDK4 (NM_000075)	F:ATGGCTACCTCTCGATATGAGC
	R:CATTGGGGACTCTCACACTCT
CDK6 (NM_001145306)	F:GCTGACCAGCAGTACGAATG
	R:GCACACATCAAACAACCTGACC
CyclinD1 (NM_053056)	F:GCTGCGAAGTGGAAACCATC
	R:CCTCCTTCTGCACACATTTGAA
CyclinE1 (NM_004864)	F:ACCTGCACCTGCGTATCTCT
	R:CGGACGAAGATTCTGCCAG
GAPDH (NM_001256799)	F:ACAACTTTGGTATCGTGGAAGG
	R:GCCATCACGCCACAGTTTC

### Immunohistochemistry

Immunohistochemistry was performed on 5-μm-thick paraffin sections. Monoclonal antibody against ZWINT was used at a 1:50 dilution (HAP022264, Sigma). After dewaxing, immunostaining permeation solution was applied to the samples. The presence of brown particles in the nucleus and cytoplasm indicated positive staining.

### Chromatin Immunoprecipitation

The EZ-ChIPTM Chromatin Immunoprecipitation Kit (Millipore, Billerica, MA, United States) was used to perform ChIP assays following the manufacturer’s instructions. Rabbit anti-FLAG (Cell Signaling Technology, United States), anti-RNA polymerase II antibodies (Abcam, United Kingdom) and related rabbit-IgG (Cell Signaling Technology, United States) was applied as controls.

### Cell Culture

The pancreatic cancer cell lines (AsPC-1, BxPC-3, MIA PaCa-2, SW 1990, PANC-1 and PANC03.27) and the HPDE normal pancreatic cell line were purchased from the ATCC. MIA PaCa-2, SW 1990, and PANC-1 cells were cultured in conventional DMEM medium (GIBCO, United States) containing 10% fetal bovine serum (BI, United States). AsPC-1, PANC03.27 and HPDE cells were cultured in RPMI 1640 medium (GIBCO) containing 10% fetal bovine serum. Cells were cultured at 37°C, 5% CO_2_ with saturated humidity.

### Cell Transfection and Lentivirus Infection

The liposome method (Lipo 3000 transfection kit, Invitrogen) was used to transfect small interfering RNA (siRNA) into PANC-1 and MIA PaCa-2 cell lines (siRNA sequences were shown in Table 2). Cells were harvested at 48 h and analyzed by RT-PCR and western blotting to determine the effect of siRNA transfection. Table 2 shows the siRNA sequences of ZWINT, p53, p21, and that of the negative control (Ruibio, Guangzhou, China). The virus-mediated transfection and oncogene overexpression were applied to infect PANC-1 and MIA PaCa-2 cell lines. Cells were cultured in flasks at a density of 2×10^4^ cells/culture flask the day before infection. After overnight culture, the medium was replaced with DMEM (without serum) containing lentivirus (MOI = 25) at 37°C. After 8–12  h, the medium was replaced with complete medium.

**TABLE 2:** The siRNA sequence used in the transfection assay.

Gene	sequence (5′→3)
si-ZWINT#1	GCA​CGT​AGA​GGC​CAT​CAA​A
si-ZWINT#2	GAA​CCA​GTG​GCA​GCT​ACA​A
si-P53	GTA​CCA​CCA​TCC​ACT​ACA​A
si-P21	GAT​GGA​ACT​TCG​ACT​TTG​T

### Western Blot

Cells were lysed using RIPA buffer for 30 min at 4°C, followed by centrifugation at 12,000×*g* at 4 °C for 30 min. The BCA method was used to determine the protein concentration of supernatants. Denatured protein samples were separated by 10% sodium dodecyl sulfate-polyacrylamide gel electrophoresis (SDS-PAGE) and transferred to polyvinylidene difluoride (PVDF) membranes (Millipore, United States). The membrane was blocked with 5% milk or bovine serum albumin (BSA) for 2 h and then incubated with primary antibodies at 4 °C overnight. After washes with Tris-buffered saline (TBST), membranes were incubated with secondary antibody (1:2000, abclone, Wuhan, China) for 2 h, followed by washes with TBST. The ECL reagent (Thermo Scientific ECL) was used to visualize bands. The experiment was performed three times.

### Cell Counting Kit-8

Cell proliferation ability was assessed by CCK-8 (Dojindo Molecular Technologies, Inc., Kyushu, Japan) based on the manufacturer’s instructions. Cells were seeded into 96-well culture plates at 2×10^3^ cells per well the day before transfection or infection. The viability of pancreatic cancer cells was evaluated from five replicates in three independent experiments after indicated treatments for 6, 24, 48, and 72 h.

### Cell Cycle Analysis

Cells were fixed with 70% ethanol overnight at 4 °C. A staining working solution of 500 μl PI/RNase (PI:RNaseA prepared at 9:1) (KeyGen Biotech, Nanjing, China) was added when centrifugation was used to remove the ethanol in the following day. Cells were stained for 3 min at 4°C in the dark. Stained cells were then examined using a flow cytometer (US BD company).

### Immunoprecipitation and Co-Immunoprecipitation

Anti-ZWINT antibody (1 μg) (Sigma: HAP022264) or anti-rabbit IgG antibody (Abcam: ab190492) was added to protein lysates (500 μg) and the samples were incubated at 4 °C overnight. Next, 40 μl protein A/G PLUS-Agarose beads (Santa Cruz: sc-2003) was added and samples were incubated at 4°C for 6 h. After adding 2 × loading buffer, the immunoprecipitated protein complex was boiled and denatured. Samples were then analyzed by western blot. 1 μg of the anti-ZWINT primary antibody was employed to perform the same approach, so as to precipitate p53 and MDM2, and western blotting with the anti-p53 and anti-MDM2 primary antibody was conducted to detect the precipitated protein.

### Immunofluorescence

Cells on glass coverslips were fixed with 4% paraformaldehyde for 20 min at room temperature. After 15 min incubation with immunostaining permeation solution (Triton X-100, P0096, Beyotime), PBS containing 5% BSA was used to block cells for 30 min. Cells were then incubated with primary antibody against ZWINT (1:50, diluted in blocking buffer) at 4 °C overnight and then with Alexa Fluor 488-goat anti-mouse IgG (1:100) secondary antibody for 2 h. Cells were then rinsed with PBS for 15 min, and DAPI was applied for 5 min for nuclei staining. In some experiments, cells were incubated with anti-p53 and anti-p21 primary antibody and the anti-ZWINT primary antibody (1:50, Sigma) simultaneously. Alexa Fluor 488-goat anti-mouse and Alexa Fluor 594-goat anti-mouse were used as secondary antibodies.

### Nude Mouse Xenograft Model

Twelve 4-week-old female BALB/cA-nu nude mice were obtained from Beijing Huafukang Biosciences (Beijing, China) and kept in pathogen-free conditions. PANC-1 cells (5 × 10^6^) expressing either ZWINT or control vector in 150 μl of PBS were subcutaneously injected into the left flank of mice (*n* = 5 per group). The rate of tumor formation, number of tumors, tumor diameter, tumor mass and mouse body weight were measured every 7 days. Tumor volume was calculated as *V* = 1/2 × L × W^2^. Mice were killed after 37 days. Tumors were fixed in 4% paraformaldehyde and embedded in paraffin.

### Statistical Analysis

The *x* ^*2*^ test and Fisher’s exact probability tests were used to analyze data, and the single-element analysis of variance was employed to measure the data. The *t* test was used to determine statistical variations between two groups, and more than two groups were compared using the one-way analysis of variance analysis. A two-tailed *p* value of <0.05 was regarded statistically significant.

## Results

### ZWINT is Upregulated in Pancreatic Cancer

**FIGURE 1:**
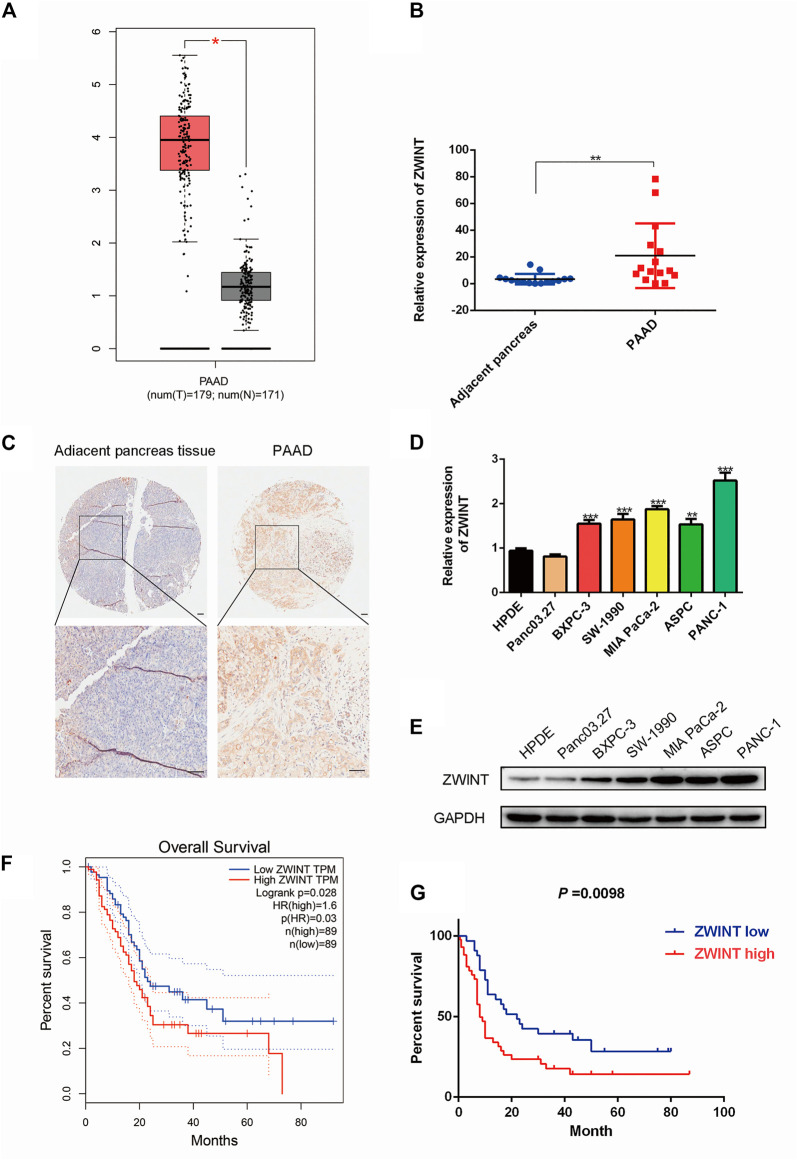
ZWINT was upregulated in pancreatic cancer. **(A)**. ZWINT expression in TCGA-LIHC database. **(B)** ZWINT expression in 16 PC tissue samples and paired normal tissue samples by q-RT-PCR. **(C)** ZWINT expression in tissue microarrays, including 92 pairs of PC samples and adjacent normal tissues. **(D, E)** q-RT-PCR and western-blot quantitatively analysis of ZWINT expression in normal and PC cell lines. **(F)** Data in the TCGA database showed the overall survival of the two groups of patients with high (*n* = 60, red line) or low (*n* = 32, blue line) PC expression in colon cancer tissues. **(G)** Kaplan–Meier representation of the overall survival of the two groups of patients with high (*n* = 60, red line) or low (*n* = 32, blue line) ZWINT expression in our cohort. **(H)** ROC analysis of the correlation of ZWINT expression and survival time. All data are the mean ± SD of three independent experiments. *p < 0.05.

To determine the role of ZWINT in pancreatic cancer tumorigenesis, a bioinformation analysis was performed using TCGA database. The results indicated that ZWINT was significantly upregulated in pancreatic cancer tissue (Figure 1A). We next performed qRT-PCR assay using 16 pairs of PC and adjacent non-tumor tissues and found that ZWINT mRNA was highly expressed in PC tissues (Figure 1B). ZWINT was further examined in a tissue microarray that included 92 paired PC samples. The average expression of ZWINT was increased in PC tissues compared with levels in nearby non-tumor tissues (Figure 1C). The positive ZWINT expression rate was 65.2% (60/92) in PC tissue samples. Further analyses of PC cell lines by qPCR and western blot assay demonstrated that ZWINT mRNA and protein level was upregulated in PC cell lines (Figures 1D,E). Based on the GEPIA (http://gepia.cancer-pku.cn/) and survival data from the cohort, Kaplan-Meier analysis, ROC analysis results indicated that ZWINT expression was negatively correlated with PC patient overall survival (Figures 1F,G). We further analyzed the relationship between ZWINT expression and clinicopathological features in PC tissue samples (Table 3). ZWINT expression was positively related to tumor size, differentiation and Vessel invasion in PC tissues. Together these results indicate that low ZWINT expression is related to progression and poor prognosis in human PC.

**TABLE 3:** The General clinical pathological characteristics of pancreatic cancer patients.

Clinical epidemiology and clinicopathologic feature	ZWINT			*p value*
	N	Low expression	High expression	
All cases	92	32	60	
Age				0.0871
≤60	25	5	20	
>60	67	27	40	
Gender				0.2586
Male	58	23	35	
Female	34	9	25	
Diameter of tumor				0.0330
≤3	29	15	14	
>3	63	17	46	
Tumor differentiation				0.0387
Well/moderate	30	15	15	
Poor	62	17	45	
Pathological T				0.6620
T1/T2	51	19	32	
T3/T4	41	13	28	
Lymph node metastasis				0.5045
N0 (negative)	55	21	34	
N1 (positive)	37	11	26	
Vessel invasion				0.0121
Negative	69	29	40	
Positive	23	3	20	

Note: Low/high by the sample median, used fisher’s exact test.

*p < 05 was considered to be statistically significant.

### ZWINT Enhances PC Cell Proliferation and Induces G1/S Phase Transition *in Vitro*

**FIGURE 2:**
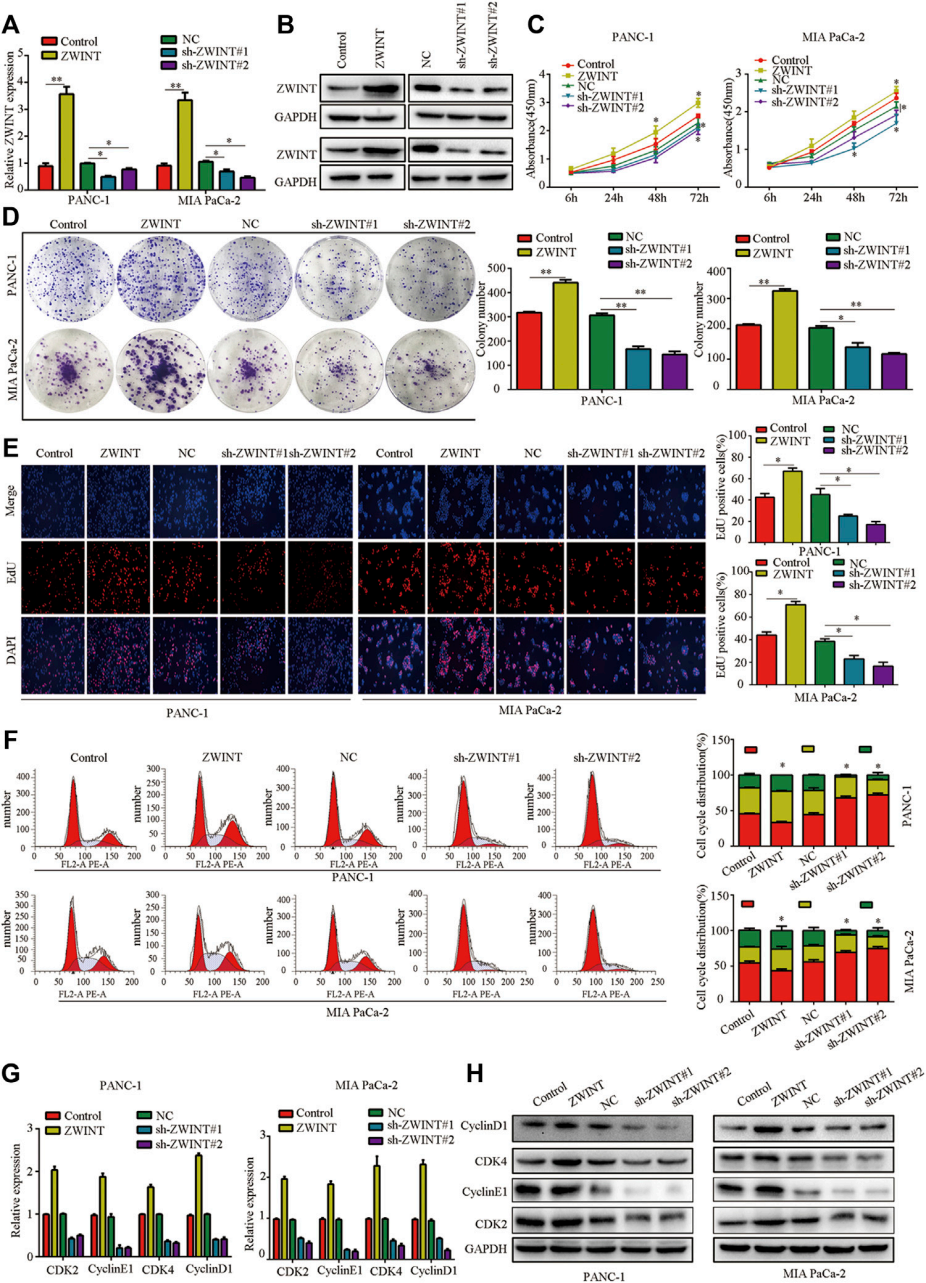
ZWINT enhances PC cell proliferation induces G1/S phase transition in vitro. **(A,B)** q-RT-PCR and western-blots confirmed the knockdown and overexpressed of ZWINT. **(C,D)** CCK-8 and plated formation analysis of the growth ability of PC cell. **(E)** EDU assay was performed to analysis the proliferation of PC cell. **(F)** Cell cycle analysis showed the effects of ZWINT on PC cell cycle regulation. **(G-H)**. q-RT-PCR and western-blots analysis of the cell cycle associated genes expression.

To clarify the role of ZWINT in regulating PC cell phenotype, we performed loss-of-function and gain-of-function assays in PC cells (Figures 2A,B). CCK-8 and colony formation assays showed that PC cell proliferation was reduced by ZWINT knockdown, while increased proliferation was observed in cells with overexpression of ZWINT (Figures 2C,D). EDU assay also showed that ZWINT overexpression significantly promoted PC cell proliferation (Figure 2E). We next evaluated the role of ZWINT in cell cycle regulation of PC cells using flow cytometry assays. We detected a significant increase in the G1/S phase population of ZWINT-depleted PC cells compared with the control group (Figure 2F). qPCR and western blot further demonstrated that ZWINT promoted cell cycle–associated gene and protein expression (Figures 2G,H). These findings indicate PC cell proliferation induces G1/S phase arrest was enhanced by ZWINT, making contribution to PC progression.

### Hypoxia Micro-Environment Induce ZWINT Upregulation

**FIGURE 3:**
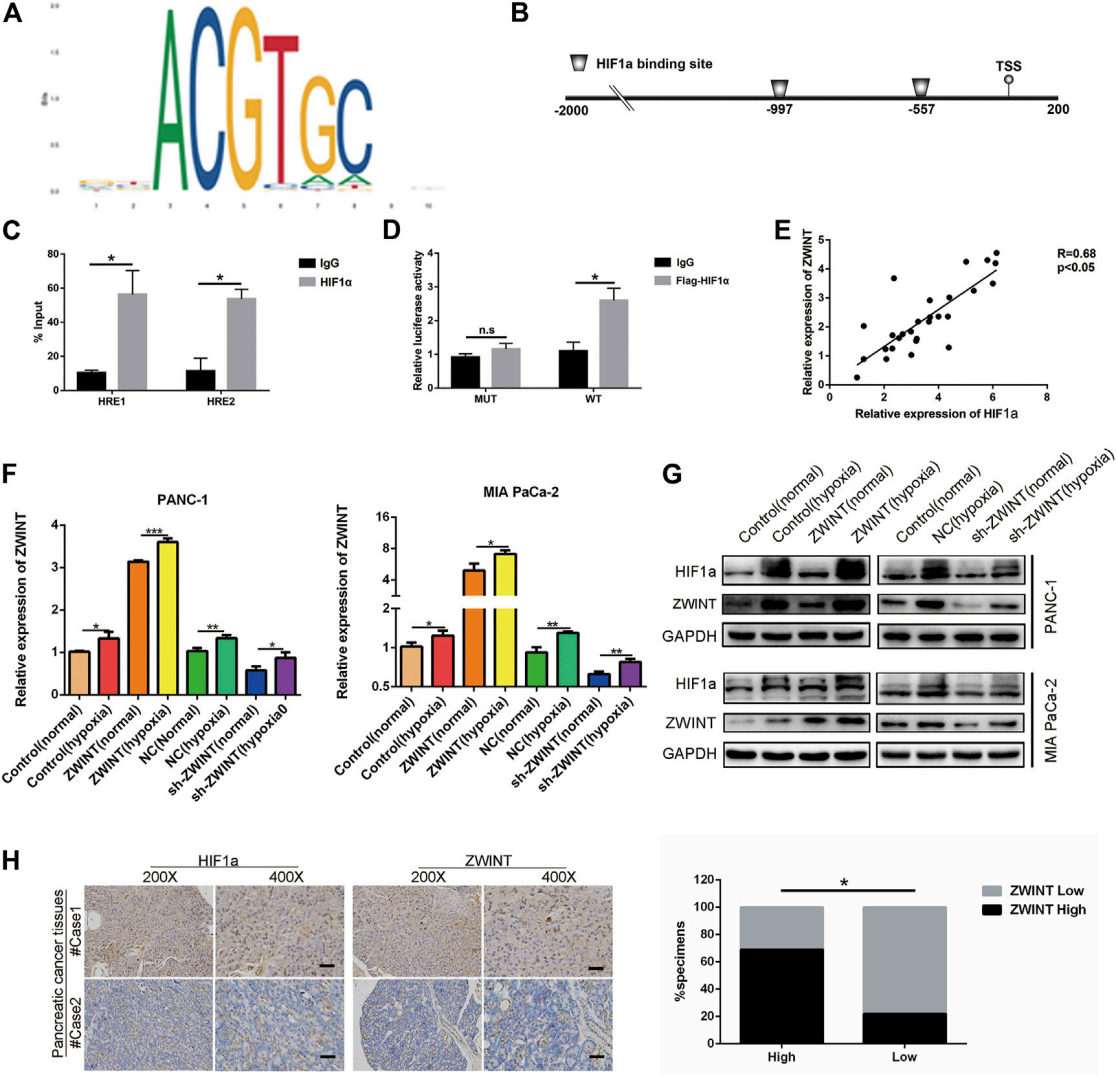
ZWINT was a direct transcriptional target of HIF-1α. **(A)** Bioinformation analysis the promoter sequence region. **(B)** Schematic diagram indicated the potential promoter binding region. **(C)** HEK293 cells were transfected with pGL3 reporter vector containing ZWINT wide-type promoter (HRE-WT), or mutant-type promoter (HRE-MUT), respectively. Those transfected cells were further treated under normoxia or hypoxia. After 48 h, firefly luciferase activity was detected and normalized by renilla activity. **(D)** ChIP assay with anti-HIF-1α antibody was performed to verify the binding between HIF-1α and two HREs in ZWINT promoter in PANC-1 cells. **(E)** Pearson correlation analysis was performed to test the correlation between ZWINT and HIF1α. **(F, G)** The mRNA and protein expression levels of ZWINT and HIF-1α in PC cells were measured after culturing under normoxia, hypoxia (CoCl_2_, 100 μM)) for 24 h by qRT-PCR and Western blot. **(H)** q-RT-PCR and IHC analysis the correlation of HIF-1α and ZWINT in the pancreatic cancer tissues. Data shown are mean ± SD (*n* = 3). (*p < 0.05, **p < 0.01, ***p < 0.001).

We next explored the elements that induced high ZWINT expression in PC. Promoter sequence analysis tools (UCSC and JASPAR) (Figure 3A) were used to inspect the genomic region (∼2 kb upstream) upstream of the ZWINT gene. The results showed two putative HIF1α binding sites within the ZWINT promoter area (Figure 3B). We next performed ChIP-qPCR assay and confirmed that HIF1α directly bound to the chromatin fragments of the two predicted ZWINT promoter areas (Figure 3C). Luciferase assays also demonstrated that wild-type (WT) ZWINT promoter activity was significantly upregulated by HIF1α, while the mutant construct with mutations in the putative HIF1α-binding sequences showed no changes in response to HIF1α expression (Figure 3D). We performed q-RT-PCR analysis for ZWINT and HIF1α mRNA in PC tissues and the pearson analysis results revealed a positive correlation (Figure 3E). After 24 h treatment of hypoxia or the chemical inducer CoCl_2_, the expression of ZWINT in PANC-1 and MIA PaCa-2 cells was increased on basis of upregulated HIF1α expression in the mRNA and protein level (Figures 3F,G). Immunohistochemistry of HIF1α and ZWINT in PC tissues showed that HIF1α and ZWINT expressions were positively correlated (Figure 3H). Together these data indicate that ZWINT is transcriptionally upregulated by HIF1α in the hypoxic tumor micro-environment. Subsequently, functional experiments were performed to measure the proliferation of PC cells in the condition of hypoxia compared with normoxia. The results suggested that hypoxia-mediated ZWINT overexpression significantly promoted cell proliferation compared with normoxia (Supplementary Figure S1).

### ZWINT Negatively Regulates the p53/p21 Signal Pathway

**FIGURE 4:**
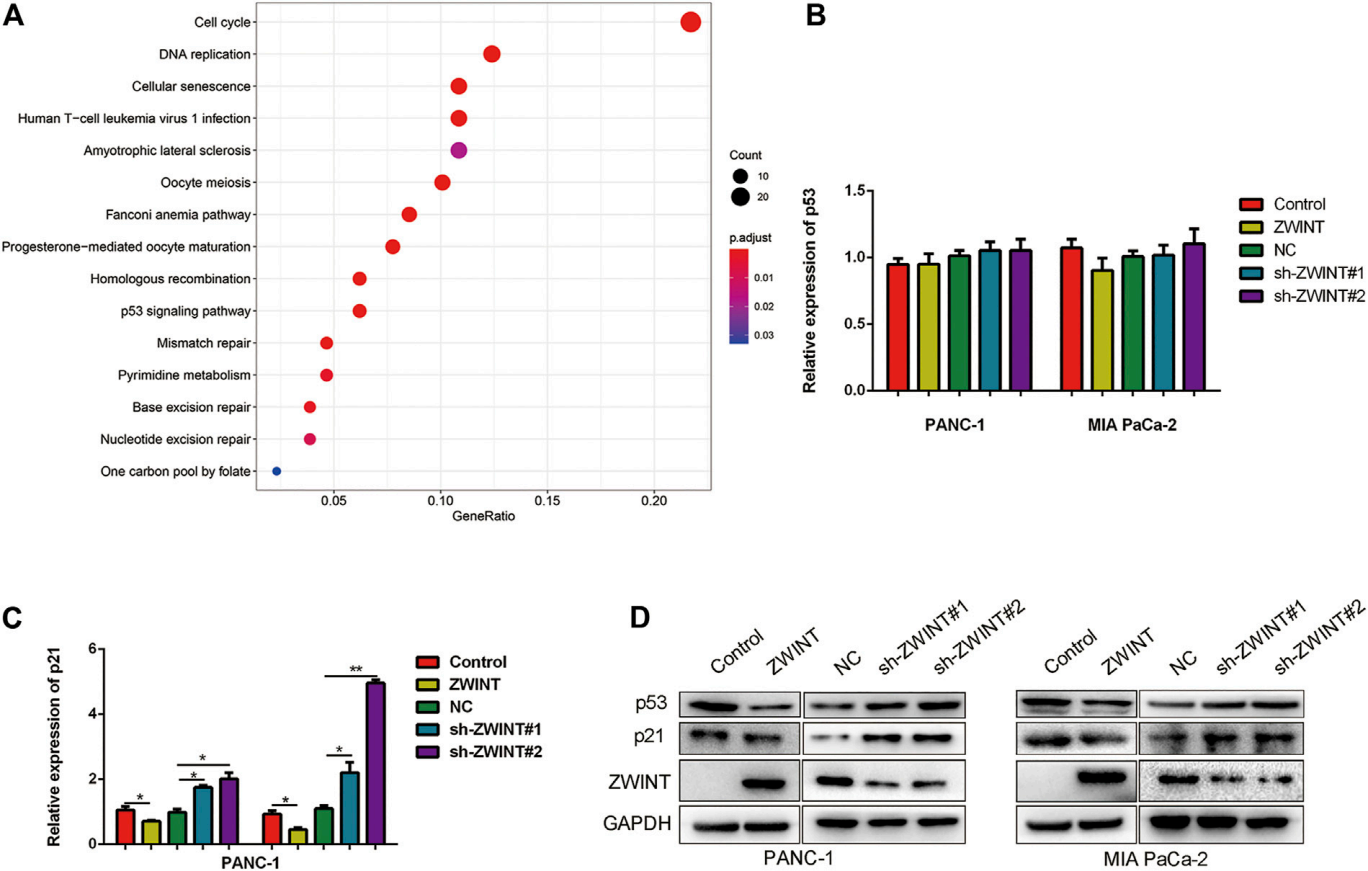
ZWINT interacted and regulated p53/p21 signal pathway. **(A)** Bioinformatic analysis the downstream pathway of ZWINT based on the TCGA databse. **(B, C)** q-RT-PCR and western blot analysis the expression of p53 and p21 in mRNA level. **(D)** Western blot analysis the expression of p53 and p21 in protein level.

To elucidate the downstream signal pathway of ZWINT, we analyzed the potential targeted pathways using the TCGA database. The results indicated that ZWINT likely regulates the cell cycle, DNA replication, and the p53 signaling pathway (Figure 4A). As ZWINT showed cell cycle regulation activity and p53 is a key regulator of the cell cycle, we next examined whether ZWINT affected p53 expression and activity. We first performed qRT-PCR to analyze p53 and p21 mRNA expression. ZWINT had no effect on p53 mRNA expression level and p21 expression was significantly upregulated in the ZWINT knockdown group (Figures 4B,C). Western blot assay revealed that p53 and p21 were significantly downregulated in ZWINT-overexpressing pancreatic cancer cells (Figure 4D).

### ZWINT Decreases p53 Expression by Promoting its Ubiquitination *via* Enhancing MDM2 Levels

**FIGURE 5:**
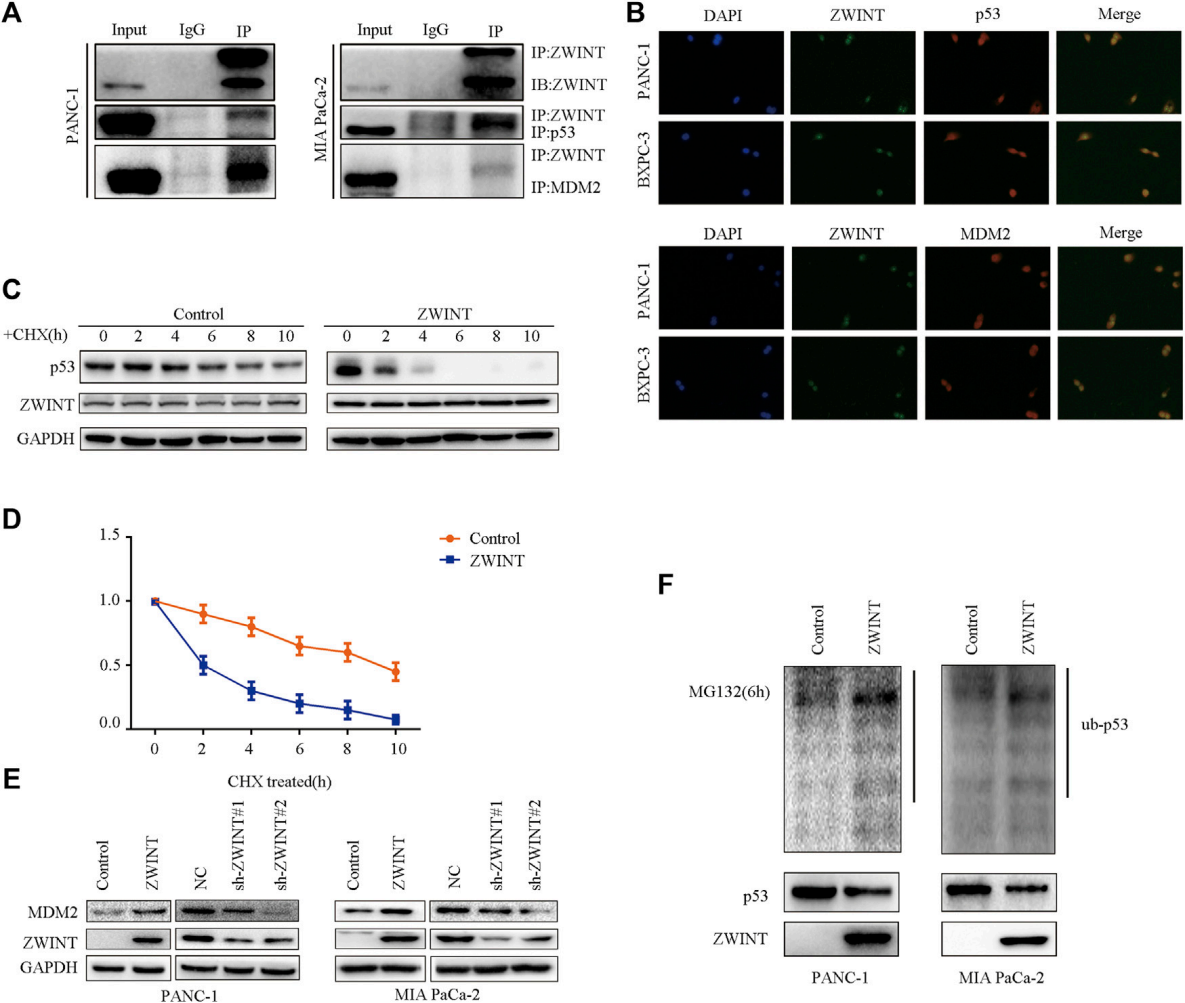
ZWINT decreases p53 expression by promoting its ubiquitination via enhancing MDM2 function. **(A)** Immunoprecipitation analysis the interaction of ZWINT with p53 and MDM2. **(B)** Immunofluorescence analysis the interaction of ZWINT with p53 and MDM2. **(C)** Western blot analysis MDM2 expression in the ZWINT upregulated and knockdown group. **(D–E)** The protein stability analysis the p53 stability in the ZWINT upregulated group. **(F)** The ubiquitination assay analysis the p53 ubiquitination level in the ZWINT upregulated group.

Our results showed that ZWINT regulated p53 protein level. As p53 protein levels are regulated by MDM2-mediated ubiquitination, we further examined whether ZWINT interacted with MDM2 and p53 to affect p53 ubiquitination. We first analyzed the interactions between ZWINT, p53, and MDM2. Immunoprecipitation and immunofluorescence analysis indicated that ZWINT binds with and co-localizes with p53 and MDM2 (Figures 5A,B). Subsequently, western blot analysis showed that MDM2 expression was increased by the overexpression of ZWINT, while MDM2 levels were decreased by ZWINT knockdown (Figure 5C). We next examined whether ZWINT affect p53 ubiquitination and stability. Protein stability assay indicated that ZWINT significantly inhibited p53 stability and ubiquitination assay demonstrated that p53 ubiquitination was significantly increased in the ZWINT-upregulated group (Figures 5D–F). These results suggest that ZWINT promoting the effect of MDM2 and influences the ubiquitination, stability and expression level of p53 by decreasing the expression level of MDM2.

### ZWINT Exerts Proliferation Effects by Directly Promoting the Transcription of p21

**FIGURE 6:**
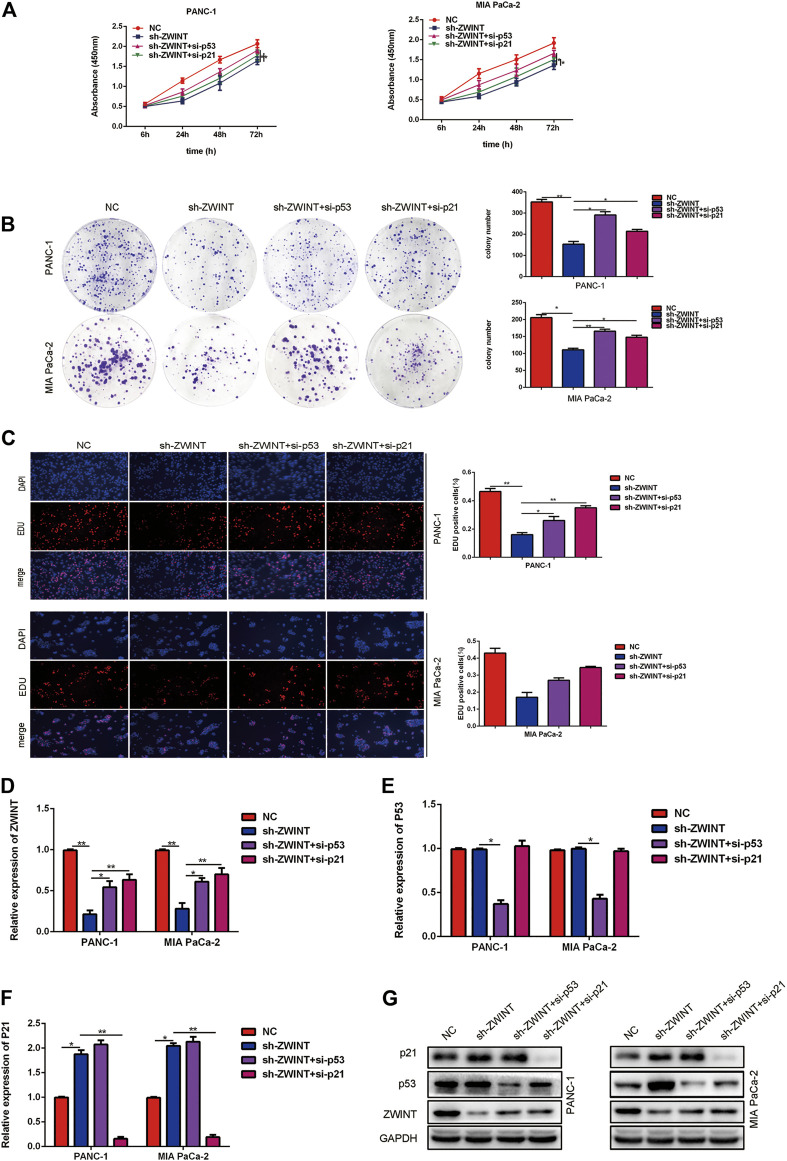
ZWINT exerted proliferation effects by directly promoting the transcription of p21. CCK-8**(A)**, plate formation**(B)** and EDU**(C)** and assay analysis the proliferation of the cells in the ZWINT knockdown transfected with p53 or p21 siRNA. **(D–G)** qRT-PCR and western blot assays analysis the ZWINT, p53 and p21 expression of the cells in the ZWINT knockdown transfected with p53 or p21 siRNA.

To investigate whether p53/p21 is necessary for ZWINT regulation of PC cell proliferation and cell cycle, we transfected p53 and p21 siRNA into ZWINT knockdown PC cells. CCK-8, colony formation assay and EDU proliferation assays showed that proliferation in ZWINT knockdown cells was enhanced upon p53 or p21 silencing (Figures 6A–C). qRT-PCR and western blot assays showed that p53 or p21 inhibition in PC cells abolished the inhibition of the expression of p53 and p21 reduced by ZWINT knockdown (Figures 6D–G).

### ZWINT Promotes Pancreatic Cancer Growth *in Vivo*

**FIGURE 7:**
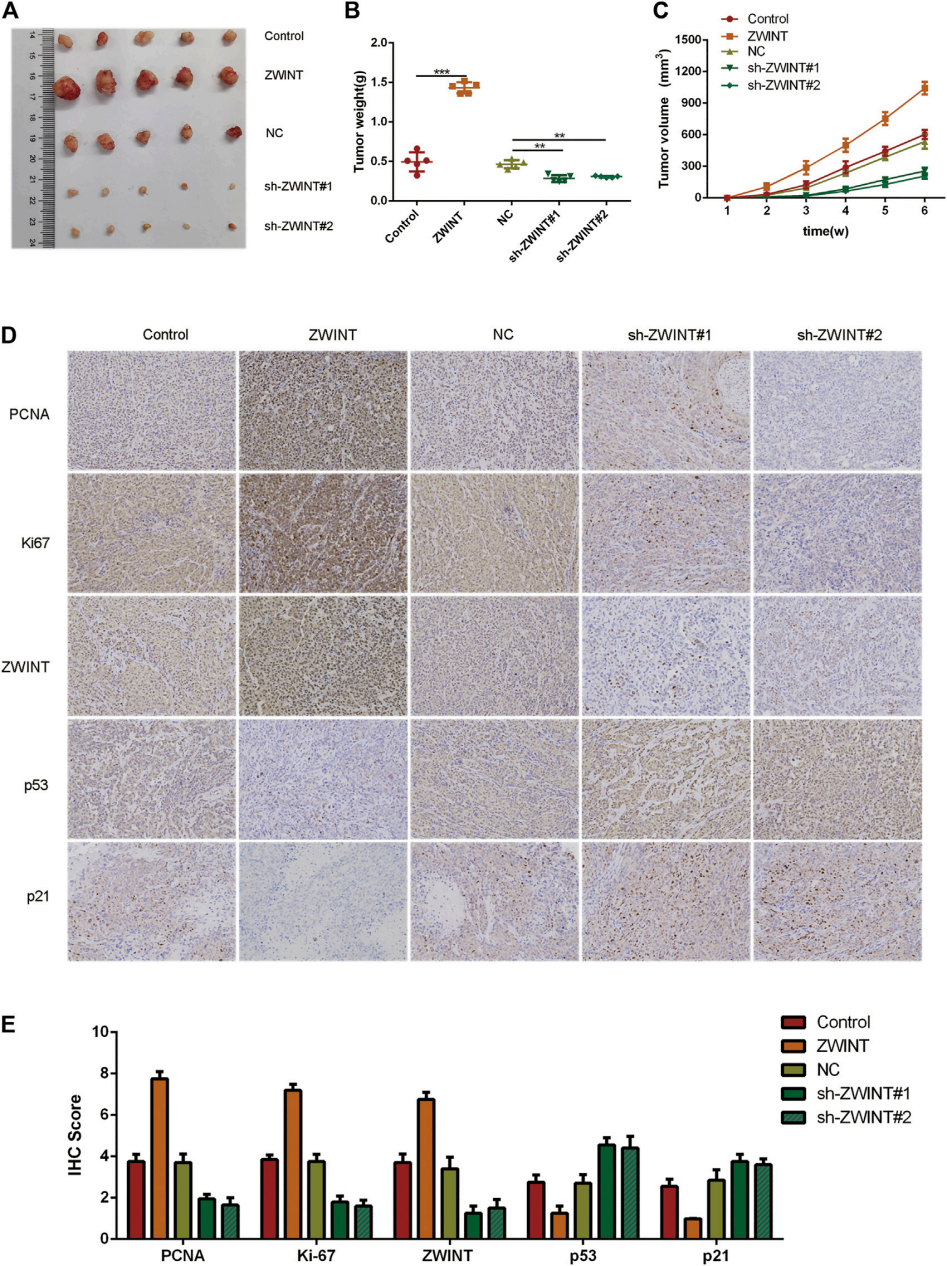
ZWINT promoted pancreatic cancer growth *in vivo*. a Represent pictures of tumor formation of xenograft of PANC-1 **(A)** cells in nude mice (*n* = 5). **(B, C)** Weight and volume of tumors in the different group mice. **(D–E)** Representative images of KI-67 and PCNA staining of the xenograft. *p < 0.05; **p < 0.01.

To determine whether tumor growth is affected by ZWINT *in vivo*, we established a xenograft mouse model by subcutaneously injecting pancreatic cancer cells overexpressing ZWINT or controls into mice (*n* = 5, each group). After 6 weeks, mice were killed and tumor samples were harvested (Figure 7A). Tumor weight (Figure 7B) and volume (Figure 7C) from cells with knockdown of ZWINT were reduced compared with controls. Immunohistochemistry showed that Ki-67 and PCNA were highly expressed in tumors from the ZWINT upregulated group and downregulated in the knockdown group (Figure 7D). Together these data indicate that ZWINT promoted pancreatic cancer growth *in vivo*.

## Discussion

In this study, we demonstrated that ZWINT, a centromere complex component, was significantly upregulated in PC tumor tissues and high expression of ZWINT was related to poor outcome in PC. ZWINT was recently reported to be an oncoprotein in multiple cancer types, including glioblastoma, breast cancer, lung adenocarcinoma. Kim JH found that ZWINT was upregulated in pancreatic cancer and promoted pancreatic cancer progression. however, the precise mechanism of its regulation was unknown (Kim et al., 2020). In this study, we focused on the mechanism of ZWINT, the interacting proteins and the downstream pathways involved in tumor regulation. Functional studies revealed that ZWINT promoted pancreatic cancer cell proliferation and cell cycle progression *in vitro* and enhanced tumor development *in vivo*. These findings suggest that ZWINT may promote PC progress.

To clarify the mechanisms by which ZWINT promotes pancreatic cancer progression, we analyzed the pathways associated with ZWINT using the TCGA database. The cell cycle and p53 signal pathway were the core targets regulator in ZWINT function. Consistent with the previous results, ZWINT could positively drive the pancreatic cancer cell cycle turning over. Abnormal cell cycle frequently occurs in cancer cells, resulting in accelerated cell division and growth in an uncontrollable rate and leading to tumor compression surrounding blood vessels and hypoxic microenvironment (Geismann and Arlt, 2020; Tao et al., 2021).

We further demonstrated that p53 expression and activity were significantly upregulated in ZWINT-knockdown pancreatic cancer cells. p53 is an upstream regulator of p21, and aberrant p53 expression and/or activity is a significant molecular hallmark of cancer (Amirinejad et al., 2020; Mansilla et al., 2020). p53 expression is typically downregulated in patients with wild-type p53, emphasizing the significance of its inhibited activity in cancer progression (Xu et al., 2021). Multiple studies have revealed various post-transcriptional modifications and regulation of p53 including phosphorylation, ubiquitination, and acetylation (Gencel-Augusto and Lozano, 2020). Ubiquitination-mediated proteasomal degradation is significant to stabilize p53 protein in post-translation (Zhou G. et al., 2020), while ubiquitination also plays role in p53 protein activation and nuclear location. Acetylation and phosphorylation play critical functions in enhancing p53 binding to its target genes (Yogosawa and Yoshida, 2018), and the cross-talk among these modifications may influence regulation of downstream target genes. Hence, according to our outcomes, the important role of p53 transcriptional regulation in the maintenance of the homeostasis of p53 expression is showed. Our results suggest that the ZWINT/p53 axis plays a key role in cell cycle regulation, cell proliferation, and tumorigenesis in PC. We also found that MDM2, the E3 ubiquitin ligase for p53 (Karakostis et al., 2020), interacts with ZWINT and we speculate that ZWINT interacts with MDM2 to influence the ubiquitination and degradation of p53. Further investigation shall be made to explore the precise mechanism by which p53 expression is regulated by ZWINT.

The stability of p53 is carefully regulated by the ubiquitin-proteasome system and p53 levels are finely tuned to respond to stressful conditions (Leu et al., 2020). Ubiquitination plays a role in the regulation of p53 expression for the regulation of cell cycle progression (Si et al., 2021). We examined the mechanism by which ZWINT regulated p53 expression and found that ZWINT inhibited p53 only at the protein level. Therefore, we speculate that ZWINT regulates p53 expression at the post-translation level. However, post-translation modifications of p53 including phosphorylation, ubiquitylation, acetylation can affect p53 expression, nuclear location, and transcriptionally activity (Sun et al., 2021).

To be different, according to our current research, our results suggest that ZWINT interacted with and restrained MDM2, to inhibit p53 protein stability by enhancing its degradation.

p21, a target gene of p53, inhibits cyclin D1 and CDK, resulting in G1/S arrest (Zhou et al., 2020b; Qi et al., 2021). Our results showed that p21 expression and transcriptional activity were inhibited in ZWINT knockdown pancreatic cancer cells. To our knowledge, this is the first data showing that ZWINT contributes to PC cell progression by regulating p53/p21 signaling.

Our data showed that hypoxia promoted the expression of ZWINT in pancreatic cancer, and ZWINT promoted pancreatic cancer proliferation *in vitro* and *in vivo*. In addition, ZWINT could inhibited p21 transcriptional activity by interacting with p53 mediated pancreatic progress.

## Data Availability

The raw data supporting the conclusion of this article will be made available by the authors, without undue reservation.

## Abbreviations

CCK-8, Cell Counting Kit-8; ChIP, Chromatin immunoprecipitation; PC, pancreatic carcinoma; mZWINT-1, ZW10-interacting kinetochore protein 1.
