# Supplementary figures and images for: Hypoxia-Induced ZWINT Mediates Pancreatic Cancer Proliferation by Interacting With p53/p21
Source: Front Cell Dev Biol. 2021 Nov 24;9:682131. doi: 10.3389/fcell.2021.682131 (PMC8652205; doi:10.3389/fcell.2021.682131)

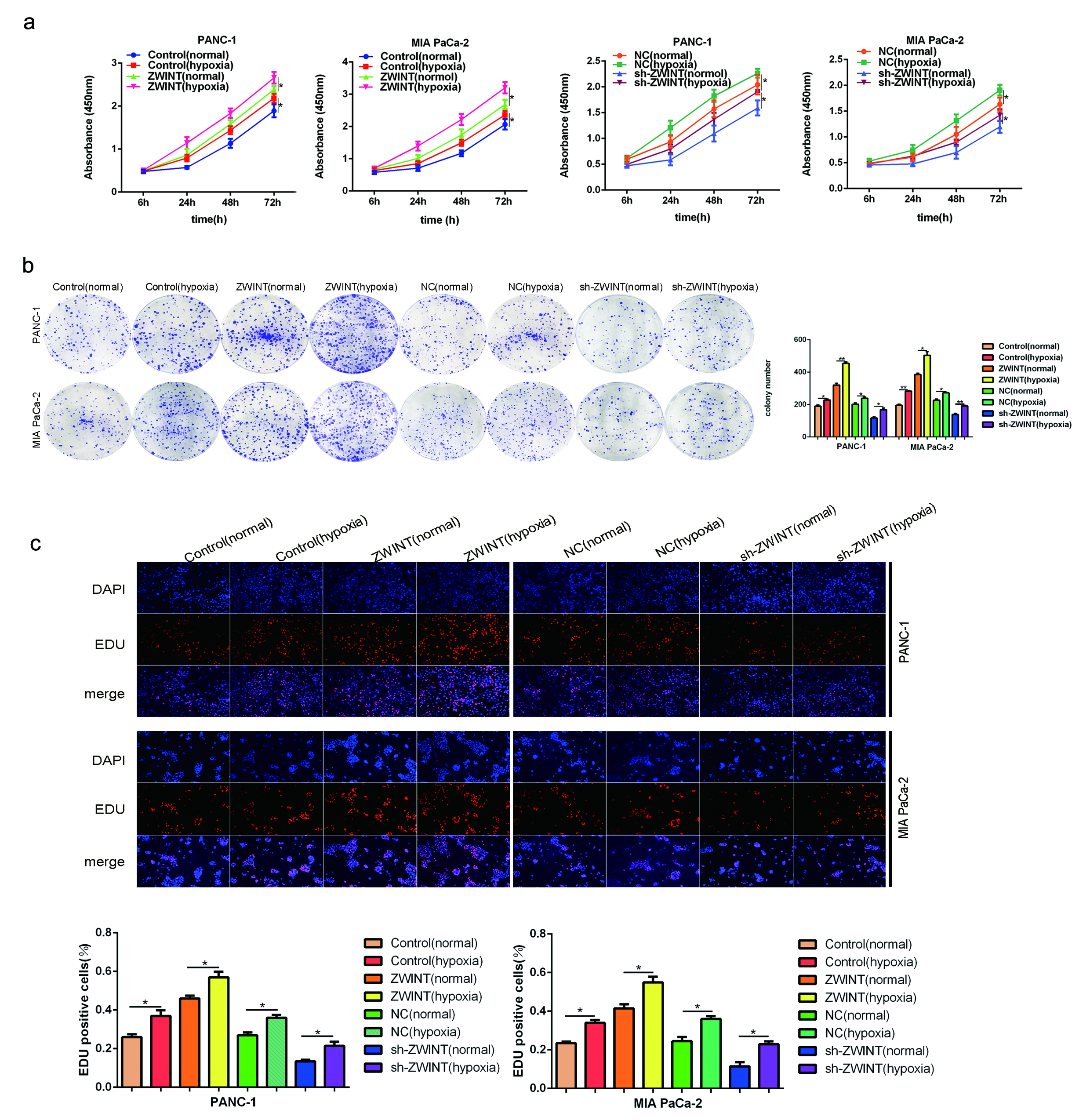

Supplement: Supplementary file 1 [file Image1.TIF]
